# The mechanisms, regulations, and functions of histone lysine crotonylation

**DOI:** 10.1038/s41420-024-01830-w

**Published:** 2024-02-08

**Authors:** Jing-yi Xie, Jie Ju, Ping Zhou, Hao Chen, Shao-cong Wang, Kai Wang, Tao Wang, Xin-zhe Chen, Yan-chun Chen, Kun Wang

**Affiliations:** 1 Institute for Translational Medicine, The Affiliated Hospital of Qingdao University, College of Medicine, Qingdao University, Qingdao, 266021 China; 2 Department of Physiology, School of Basic Medical Sciences, Shandong Second Medical University, Weifang, 261053 China; 3 State Key Laboratory of Cardiovascular Disease, Heart Failure center, Fuwai Hospital, National Center for Cardiovascular Diseases, Chinese Academy of Medical Sciences, Peking Union Medical College, Beijing, 100037 China; 4 Neurologic Disorders and Regenerative Repair Laboratory, Shandong Second Medical University, Weifang, 261053 China

**Keywords:** Post-translational modifications, Acetylation

## Abstract

Histone lysine crotonylation (Kcr) is a new acylation modification first discovered in 2011, which has important biological significance for gene expression, cell development, and disease treatment. In the past over ten years, numerous signs of progress have been made in the research on the biochemistry of Kcr modification, especially a series of Kcr modification-related “reader”, “eraser”, and “writer” enzyme systems are identified. The physiological function of crotonylation and its correlation with development, heredity, and spermatogenesis have been paid more and more attention. However, the development of disease is usually associated with abnormal Kcr modification. In this review, we summarized the identification of crotonylation modification, Kcr-related enzyme system, biological functions, and diseases caused by abnormal Kcr. This knowledge supplies a theoretical basis for further exploring the function of crotonylation in the future.

## Facts

- Protein crotonylation is a non-acetylated modification discovered with the development of mass spectrometry.
- The crotonylation involves many physiological processes, and abnormal crotonylation is related to the development of some diseases.
- Targeting the process of crotonylation provides a huge research prospect for disease treatment.
- The identification of non-histone crotonylation increased the understanding of the field of protein modification and precise modulation of protein functions.

## Open questions

- Owing to crotonylation and other forms of post-translational modification sharing identical enzymes, the crosstalk of crotonylation and other forms of post-translational modification in the modulating protein function should be further studied.
- The crotonylation-related enzyme system lacks sufficient awareness, so further study should focus on the discovery and identification of crotonylation-related enzymes.
- More studies should focus on the role of crotonylation in the non-histone proteins, reveal more lysine crotonylation sites, and clarify the influences on the protein functions.

## Introduction

Post-translational modifications (PTMs) elevate the diversity of protein functions by introducing specific chemical modifications of proteins. Precursor proteins undergo a battery of post-translational modifications to become functionally mature proteins. Excision of N-formylmethionine, methionine, disulfide bond formation, shearing, and process of proteins and modification are important approaches for protein maturation. PTMs regulate not only molecular biological processes, including DNA replication, and transcription, but also various cytobiology processes, such as cell differentiation and development. The misadjustment of PTMs involves many pathology situations, such as neuropsychiatric disease, virus infection, and tissue injury. The most well-known modification type is acylation, which influences location and dynamic interactions with certain proteins, nucleic acid, lipids, and other molecules. Both histone and non-histone can be acetylated, which regulates gene expression and directly affects protein functions.

The rapid development of high-resolution liquid chromatography-tandem mass spectrometry (LC-MS/MS) has continuously enriched the modification types of PTMs over the past ten years. Crotonylation (Kcr), as one of the novel histone acylation modifications draws a lot of attention from the research scientist. These modifications are the addition of an acyl group from an acyl-CoA donor to the ε-amino group of the lysine side chain, which is similar to acylation modification but differs in hydrocarbon chain length, hydrophobicity, or charge. However, different acylation groups have chemical differences, which are classified into three groups: the hydrophobic acyl group, the polar acyl group, and the acidic acyl group based on the chemical properties of lysine modification. The hydrophobic groups of Kcr neutralize the positive charge of lysine residues, which influences the biological function of substrate proteins. The new research indicates that Kcr modification of DNA histone influences gene regulation as well as the complexity of chromatin biology. In addition, the lysine Kcr modification of non-histone proteins also plays important roles in physiological and pathological processes, which expands the understanding of Kcr modification.

Kcr, a histone acylation modification, was first described in 2011. Up to now, various histone and non-histone Kcr sites have been identified in different tissues. For example, in the human somatic and male germinal cells, the Kcr modification is detected to be enriched in the transcriptional start sites (TSS) and enhancer regions to regulate gene expression [1]. A large amount of non-histone Kcr modification was identified in the Hela cell line, and more non-histone Kcr modification was detected in different species, which influences RNA processing, nucleic acid metabolism, and gene expression [2]. The Kcr modification is 4 carbons in length, and the Kcr modification contains a carbon-carbon (C-C) π-bond, forming a unique rigid planar conformation [3]. Therefore, this review focused on the metabolic and enzyme systems related to Kcr modification, its potential roles in physiological conditions and pathological damage, and summarized and analyzed the existing reports on Kcr modification, to provide information for further investigation of croton acylation modification.

## The identification methods of Kcr modification

Protein modification analysis by mass spectrometry is the identification of post-translational modified proteins by mass spectrometry technology. Due to the addition of related chemical groups, the molecular weight of the post-translational modified proteins will increase correspondingly compared with the non-modified proteins, and mass spectrometry technology is based on this principle for the identification of protein modifications [4]. Mass spectrometry technology directly detects peptide ions to determine their molecular mass. If the peptide ion to be detected has a certain modifying group, its molecular mass will correspondingly increase the relative molecular mass of the modifying group. For peptides or proteins with known sequence and molecular mass, the type of modifying group can be determined by calculating the difference in molecular mass [4]. Thus, the identification of post-translational modification was performed. This is a traditional MS method for identifying protein modifications.

With the development of research techniques, the types and sites of post-translational modification of proteins are constantly expanded. Zhao Yingming’s team developed a novel histone modification detection method in 2011 and analyzed the MS data from the experiment an efficient PTMap algorithm in which they found 130 histone post-translational modification sites, and 67 sites were found for the first time [1, 5]. The method and discovery greatly enriched the basic data of histone modification. Among the newly identified 67 sites, 28 core histone lysine sites do not match known modifications. To study the modification types of these sites, the modified histone H2BK5 sites were specifically selected for LC-MS /MS. Besides, the Kcr antibody specifically recognized crotonylation modification, indicating that these sites were highly likely to be crotonylated [1]. Further, D4 crotonic acid was labeled and tracked using isotope labeling, and 28 newly detected sites were finally verified. This is the first time that crotonylation modification has been found in histone modification. Crotonylation is a new acylation modification of histones and non-histones, so it is important to elucidate its metabolic mechanism [1]. The chemical group modified by crotonoyl on histones was first identified as crotonoyl, and cell tests have confirmed that the main donor of crotonoyl is crotonoyl coenzyme A and crotonoyl in vitro can be effectively converted into crotonoyl coenzyme A through cell metabolism.

## Metabolic regulation of Kcr

Crotonylation of protein is the transfer of a donor crotonylation group to a lysine residue (ε-amino) in the chain of the recipient protein [6]. The production of crotonyl-CoA is regulated by various pathways. Acyl-CoA synthetase short-chain family member 2 (ACSS2) plays an important role in the conversion of crotonate into crotonyl-CoA, and the concentration of crotonyl-CoA controls the occurrence of Kcr modification. Further, the supplement of crotonate and overexpression of ACSS2 increased the content of cellular crotonyl-CoA and histone Kcr [6, 7]. In addition, glutaryl-CoA can transform into crotonyl-CoA by β-oxidation with the help of butyryl-CoA dehydrogenase (BCDH) [8]. Acyl-CoA dehydrogenase short-chain (ACADS) and acyl-CoA oxidase (ACOX3) catalyze the conversion of butyryl-CoA to crotonyl-CoA in the processes of endoderm differentiation [9]. The metabolism of lysine, hydroxylysine, and tryptophan produces crotonyl-CoA mediated by glutaryl-CoA dehydrogenase (GCDH) [10]. Besides, chromodomain–like (CDYL) as a crotonyl–coenzyme A hydratase inhibits histone crotonylation, especially the Kcr of RPA1, which plays important roles in homologous recombination DNA repair [11]. Therefore, crotonyl-CoA, crotonate, and butyrate are important sources of Kcr modification.

Despite the concentration of crotonyl-CoA being one of the factors in influence cellular histone crotonylation, recent studies have revealed that enzyme-dependent Kcr writer or eraser regulates the dynamic balance of Kcr modification. We reviewed the identification and characterization of Kcr modification-related enzymes to clarify the regulatory mechanisms of Kcr.

## The modification enzyme of histone crotonylation

A series of factors are involved in histone crotonylation regulation, which mainly plays roles as writers, erasers, and readers or other regulators of Kcr. We, therefore, summarize the enzyme that participated in the histone crotonylation in Table 1.

**Table 1:** The regulatory factors involved in the crotonylation modification.

Regulatory mode	Regulatory molecules	Crotonylation site	Reported date	Reference
Writer	P300	H3K18	2015	[6]
	MOF	H3K4, H3K9, H3K18, H3K23, H4K8, and H4K12	2017	[17]
	GCN5	H3K9, H3K14, H3K18, H3K23, and H3K27	2019	[20]
	Esa1	H4K5, H4K8, H4K12, and H4K16	2019	[20]
Eraser	SIRT1, 2, 3	H3K4	2014	[23]
	HDAC1, 2, 3	H3K4, H3K9, H3K23, H4K8, H4K12 and H3K23	2017	[7]
Reader	Taf14	H3K9	2016	[26]
	AF9	H3K9, H3K18 and H3K27	2016	[28]
	MOZ	H3K14	2016	[29]

## Kcr writers

The Kcr writers are a variety of Kcr modification enzymes. Until now, several histone acetyltransferases (HATs) that act as Kcr writers have been identified to be related to Kcr modification. MYST family, p300/ CREB-binding protein (p300/CBP) and Gcn5-related N-acetyltransferase (GNAT) are three members of the HAT family but they have been reported to catalyze Kcr by using crotonyl-CoA as substrate. The HAT-p300, a well-studied transcription co-activator, has the most promiscuous acyltransferase functions. Besides the function of acetyltransferase activity [12], p300 plays important roles in catalyzing histone Kpr [13], Kbu [13], Kcr [6]and Kbhb [14] as well as Ksucc [15] and Kglu [16]. The crystal structure of p300 in complex with propionyl-, crotonyl-, or butyryl-CoA shows that the aliphatic portions of these cofactors are bound in the lysine substrate-binding tunnel in a conformation that is incompatible with substrate transfer. The reasons for the cofactor preferred by p300 are the size of the pocket and its aliphatic nature excluding long-chain and charged acyl-CoA variants [14]. The longer the acyl chain length, the weaker the acyltransferase activity of p300, due to the restriction of substrate-assisted rearrangement and the aliphatic back pocket [14]. Histone Kcr exists in various eukaryotes including yeast, p300/CBP is one of major histone crotonyltransferase (HCT), the mutation of p300/CBP lacks histone acetyltransferase activity but reserves histone crotonyltransferase activity, which instead endogenous CBP/p300 to elevate transcriptional activity [17]. Later, the global quantitative proteomics study characterizes the p300-regulated lysine crotonylome, which reveals that p300 regulates various pathways including nonsense-mediated decay, infectious disease, and viral/eukaryotic translation pathways [18].

**Fig. 1:**
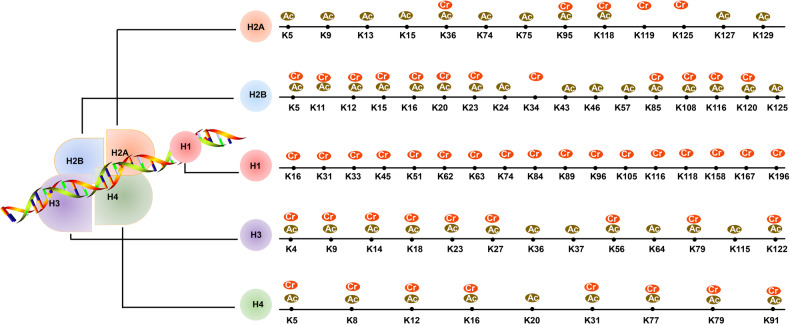
The modification sites of histone crotonylation and lysine acetylation. The Kcr and Kac modification sites of histone H1, H2A, H2B, H3, and H4 are exhibited as above.

After the identification of p300 having the HCT activity, the members of MYST family Esa1 and human homology males absent on the first (MOF) also had HCT activity. However, the crotonyltransferase activity of p300 is inefficient in vitro and needs other cellular p300 partners to enhance its crotonyltransferase activity in cells [17]. A similar situation also occurred in recombinant MOF and Esa1 in vitro, both MOF and Esa1 have low HCT activities, which indicates that they are part of a protein complex and need cofactors to enhance their activity or their activity is regulated by undiscovered modifications [19]. Gcn5 and Esa1 have crotonyltransferase activity and a study reveals that Gcn5-Ada2-Ada3 (ADA) and Esa1-Yng2-Epl1 (Piccolo NuA4) histone acetyltransferase complexes can crotonylate histones. MS analysis revealed that ADA catalyzes crotonylation at Lys residues 9, 14, 18, 23, and 27 of histone H3 and Piccolo NuA4 crotonylate Lys residues 5, 8, 12, and 16 in histone H4. The ADA composed of Gcn5 and Piccolo NuA4 composed of Esa1 is real crotonyltransferases that enhance crotonylation-dependent transcription [20]. Up to now, a lot of Kcr modification sites have been found, and we have marked the modification sites in Fig. 1.

## Kcr erasers

The decrotonylases are known as “eraser” which plays roles in the removal of covalent modification of lysine crotonylation. Several deacetylases were reported to serve as histone decrotonylase (HDCR), which is divided into four categories. The first one is NAD-dependent sirtuin family [class III SIRT1‐7], the second one is zinc‐dependent Rpd3/Hda1 family [classes I histone deacetylase (HDAC1, 2, 3 and 8)], the third one is class II [HDAC4, 5, 6, 7, 9 and 10] and the last one is class IV [HDAC11].

The researcher screened the activities of eleven human zinc-dependent lysine deacetylases against a series of fluorogenic substrates as well as kinetic evaluation and found that HDAC10 and HDAC11 have deacetylase activity at low concentrations. Moreover, the complex composed of HDAC3 and nuclear receptor co-repressor 1 has been proven to have decrotonylase activity in vitro [21]. Crotonyl group and lipoic acid modification are long-chain acylation modifications, which are deacylated by decrotonylases SIRT1, SIRT2, and SIRT1, SIRT2, SIRT3, and SIRT4 respectively [22]. The chemical proteomics approach was used to identify ‘eraser’ enzymes that recognize a lysine-4 crotonylated histone H3 (H3K4Cr) mark. SIRT1, SIRT2, and SIRT3 were identified to catalyze the hydrolysis of lysine crotonylated histone, and SIRT3 was confirmed to function as a decrotonylase to control histone Kcr dynamics and gene expression with this method employed [23]. Class I histone deacetylases (HDACs) and the Sirtuin family are the major histone decrotonylases, which play important roles in histone acetylation in mammalian cells [7]. What’s more, histone deacetylase 1 and 2 (HDAC1/2), as the catalytic core of numerous co-repressor complexes, are important histone decrotonylase enzymes. A ternary complex composed by HDAC1/CoREST1/LSD1 hydrolyses both histone H3 Lys18-acetyl (H3K18ac) and H3 Lys18-crotonyl (H3K18cr) peptide substrates. Depletion of HDAC1/2 increased global histone crotonylation and reduced total decrotonylase activity [24]. HDAC1 and HDAC3, but not HDAC2, decrotonylate non-histone proteins NPM1, and this effect can be reversed upon HDAC inhibitor TSA treatment, which indicates that deacetylases HDAC1 and HDAC3 act as decrotonylases for non-histone proteins [25].

## Kcr readers

In the cytoplasm, the content of crotonyl-CoA, the percentage of crotonyl-CoA/acetyl-CoA, as well as the homeostasis balance between crotonyltransferase and decrotonylase are important components of Kcr transformation. The researchers have found that the YEATS, bromodomain, as well as double PHD finger (DPF), are important readers of Kcr modification [2]. Transcription initiation factor TFIID subunit 14 (Taf14) maintains the normal crotonyl-CoA metabolism and Kcr readout by binding to histone H3K9cr by its crotonyllysine-binding activity via π-π-π stacking mechanism [26]. DPF or YEATS domain proteins are preferred to bind histone Kcr compared with other types of acylation. Bromodomains were reported to be involved in the acetyllysine modification of histone and it also was identified to be involved in crotonyllysine modifications. Most bromodomain only binds the shorter acetyl and propionyl tags, but the bromodomains of BRD9, CECR2, and the second bromodomain of TAF1 also identify the longer butyryl mark and even the TAF1 second bromodomain can recognize crotonyl marks [27]. Although BRD9 and TAF1 can recognize Kcr, their ability to bind to crotonylated peptides is much weaker than that of acetylated peptides [28]. In addition, the AF9 YEATS domain has a higher binding activity for crotonyl- than acetyllysine, because of the extended aromatic sandwiching cage with crotonyl-specificity arising from π-aromatic and hydrophobic interactions between crotonyl and aromatic rings. In the YEATS but not the bromodomains, these structures are conserved. At a subcellular level, crotonylated histone H3 co-localized with AF9 to regulate gene expression positively dependent on the YEATS domain [28].

MOZ, MORF, and DPF1-3 are characterized as Kac readers, all of them have DPF domain proteins. However, the DPF domain proteins were also identified as Kcr-preferential readers, especially MOZ and DPF2, which tend to enrich Kcr at 4 to 8-fold compared to Kac. The crystal structures of complex composed of MOZ DPF domain with H3K14cr, H3K14bu, and H3K14pr suggested that these histone acylations are trapped in a β2 hydrophobic “dead-end” pocket with the Kcr preference derived from the intimate encapsulation and crotonylation-sensing hydrogen bonding [29].

To sum up, we will conclude that the YEATS domain-mediated π–π–π stacking mechanism and DPF domain-mediated intimate hydrophobic ‘dead-end’ mechanism is the reader regulation of Kcr modification.

## The functions of histone crotonylation

**Fig. 2:**
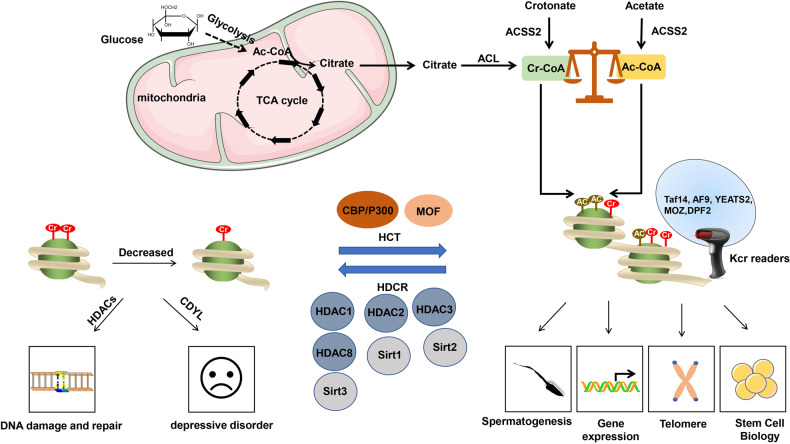
The regulation and function of histone crotonylation. The cellular concentration of acetyl-CoA and crotonyl-CoA determined the degree of histone crotonylation and acetylation. The metabolic production of acetyl-CoA and crotonyl-CoA is shown in the above pathway. The glucose was oxidized to acetyl-CoA in the mitochondrial citrate with the help of ATP-citrate lyase (ACL). Acetate and crotonate belong to short-chain fatty acids, which are converted to their cognate acyl-CoAs catalyzed by acetyl-CoA synthetase 2 (ACSS2). Crotonyl-CoA is the by-product of fatty acid and amino acid metabolism whether Crotonyl-CoA is generated from crotonate or has other ways is unknown. The higher the ratio of Crotonyl-CoA to acetyl-CoA, the easier it is to partially bind crotonyl to chromatin via acyltransferase. The histone acetyltransferases p300/CBP and MOF also have HCT activity. Class I HDACs and the members of the Sirtuins family SIRT1, 2, and 3 play important roles in the HDCR activity. Besides, the chromodomain protein CDYL inhibits histone crotonylation by converting crotonyl-CoA to b-hydroxybutyrate-CoA. YEATS2, AF9, MOZ, MORF, and DPF2 are Kcr readers that regulate various functions such as DNA damage and repair, spermatogenesis, stem cell biology, and the like.

The modification of Kcr is extensive and has been reported to be associated with a variety of physiological and pathological processes, shown as in Fig. 2.

## Regulation of gene transcription

Crotonylation of lysine (Kcr) is an evolutionarily conserved post-translational modification of histone. Histone Kcr was discovered to bind with promoters or potential enhancers to activate its transcription activity in both human somatic and mouse germ cell genomes. On the sex chromosomes, there are abundant Kcr modifications, and after completion of meiosis, Kcr modifications mark testicle-specific genes in male germ cells. Therefore, the occurrence of Kcr modification is considered to be a specific marker for the activation of sex chromosome-associated genes in male germ cells after the completion of meiosis [1]. P300 is a protein with both histone crotonyltransferase and acetyltransferase activities. Compared with histone acetyltransferase activity, the histone crotonyltransferase activity of P300 can promote transcription to a greater extent [6]. By using cellular transcriptional activation models, the research found that higher levels of Crotonyl-CoA led to changes in the level of histone crotonylation, flanking the activated gene regulatory elements and were associated with gene expression [17]. In addition, CBP/p300 mutants (p300 I1395G and CBP I1432G) lack HAT activity but have high HCT activity, which promotes transcriptional activation even though in the absence of HAT, and can be used to promote gene transcription [17]. SIRT3 inhibited the expression levels of Ptk2, Tshz3, and Wapal by reducing the histone crotonylation modification at the transcription initiation site of the target gene, suggesting that Kcr modification played a positive role in gene expression [23]. What’s more, the level of histone crotonylation is increased in the renal tissue after acute kidney injury, and the histone crotonylation in the PGC-1α and the SIRT3 elevates its expression [30]. It is worth noting that several histone Kcr sites are related to gene activation. For example, H3K18Cr is a biomarker for the activation of genes such as Il6, Gbp2, Ifit1, and Rsad2, and H2BK12Cr is a biomarker for the activation of genes post-meiotic genes, which play an important role in spermatogenesis [31].

## Transcriptional repression

Nuclear paraspeckle assembly transcript 1 (NEAT1), a long non-coding RNA, plays an important role in the progress of Alzheimer’s disease. In the early stages of AD, downregulation of NAET1 expression inhibits the frontal expression of endocytic-related genes and reduces glia-mediated Aβ clearance. The inhibition of NEAT1 down-regulates H3K27Ac and up-regulates H3K27Cr located near the TSS of endocytosis-related genes via the P300/CBP complex [32]. This suggests that H3K27Cr plays the biomarker function of transcriptional repression of endocytosis-related genes. What’s more, a recent study reveals that the gene expression and metabolic states were related to H3K9 crotonylation. When H3K9 acetylation declines and energy resources become limited, H3K9 crotonylation peaks at pro-growth genes and results in gene repression. These results indicate that H3K9Cr is related to pro-growth gene transcription repression [33].

## DNA damage and repair

The endogenous and exogenous mutagens cause DNA damage, which relies on the subsequent repair of DNA lesions to maintain genomic integrity [34]. Recently, a study shows PTM plays an important role in DNA damage and repair. HDACs are important PTM proteins in the regulation of histone Kcr in the process of DNA damage. Exposure to ionizing radiation, and ultraviolet radiation, and treatment of etoposide are important inducements of DNA damage. After the treatment of inducement of DNA damage, the level of Kcr is rapid and transient decreased and resorted at basal level [35]. HDACs are the major lysine decrotonylase and the treatment of HDACs inhibitor TSA increases the level of H3K9cr. These data reveal that the reduction of H3K9 crotonylation at DNA damage sites stimulated by various DNA damage inducers is dependent on HDAC activity [35]. However, how histone crotonylation prevents DNA damage still needs further study.

## Spermatogenesis

Spermatogenesis is a highly conserved physiological process. Obesity, diabetes, environmental chemicals, and varicocele are thought to be adverse factors in the processes of spermatogenesis. A large amount of DNA was packed into the very small-volume sperm head [36]. The protamine replaces specific histones via hyperacetylation of histone H4, which is an important event of spermatogenesis. Histone Kcr was reported to be involved in the regulation of male haploid gene expression and sperm formation after meiosis [36, 37]. Histone is enriched in the activated male germ cells and activated autosome genes after meiosis, which indicates that histone Kcr of haploid sex chromosomes is a leading element in keeping activated gene activity in the overall suppressive environment because of its resistant to transcription repressor [38]. During the processes that begin with male meiosis up to spermatids formation, the sex chromosome maintains heterochromatin status [38]. RNF-8 dependent modification, including trimethylation of H3K4, Kcr, and incorporation of histone variant H2AFZ, which regulates escaped gene activation from inactive sex chromosomes in post-meiotic spermatids. Kcr accumulation in TSS of sex-linked genes and changes in chromatin conformation are related to RNF-8-dependent epigenetic programming [38].

The chromodomain Y-like transcription co-repressor CDYL is a crotonyl-CoA hydratase that regulates the conversion of crotonyl-CoA to β-hydroxybutyryl-CoA and negatively adjusts histone Kcr. The negative regulation of histone Kcr is related to transcription repression as well as sex chromosome-linked gene reactivation in round spermatids and genome-wide histone replacement in elongating spermatids [31]. The dysregulation of histone Kcr and reduction of male fertility with a decreased epididymal sperm count and sperm cell motility were discovered in a *cydl* transgenic mice. These results reveal the function of CDYL-regulated histone Kcr modification in spermatogenesis, which is beneficial for understanding mechanisms of male reproductive physiology and failure of spermatogenesis in the azoospermia factor c (AZFC) depletion man [31].

## Telomere homeostasis

Telomeres are composed of a guanine-rich sequence and shelterin complex, which cap the end of the chromosome and hold the genomic stability [39]. Short telomeres are related to cell senescence or tumorigenesis and sufficiently long telomeres are vital to maintain self-renewal and pluripotency of pluripotent stem cells. The length of telomere is strongly correlated with the degree of reprogramming, pluripotency, and differentiation capacity of chemically induced pluripotent stem cells [40]. The small molecules’ long-term exposure reduced telomerase and enhanced telomere damage and apoptosis, which led to shortened telomeres and resisted the formation of pluripotent stem cells. In terms of mechanism, the crotonic acid inhibits the level of heterochromatic H3K9me3 and HP1α subtelomere to induce histone crotonylation and activate the expression of zinc figure and SCAN domain-containing protein 4 (Zscan4) [41]. The overexpression of Zscan4 protects the telomere against chemical drug-induced damage and keeps the length of the telomere in the process of reprogramming [41].

## Stem cell biology

The renewal of mouse embryonic stem cells depends on the production of histone crotonoylation [7]. Fang et al. found that during the differentiation of human embryonic stem cells, the endoderm increases the enzyme that induces the production of crotonoyl-CoA to induce the expression of the histone crotonoylation and endoderm genes [9]. Peroxisomal acyl-coenzyme A oxidase 3, short-chain specific acyl-CoA dehydrogenase, and ACSS2 are important regulators of crotonyl-CoA metabolism, which is significantly upregulated in endoderm differentiation [9]. Interestingly, the maintenance and transformation of pluripotent stem cells relies on the protein crotonylation revealed by the global profiling of lysine crotonylome in different pluripotent states [42]. The research has identified 3628 high-confidence crotonylation sites in 1426 proteins, which are related to proteasome function, RNA biogenesis, and central carbon metabolism [42]. Crotonylation of proteins is important for stem maintenance of pluripotent stem cells, and the atlas of protein crotonylation provides the comprehension of the change of metabolism in the cell fate transitions.

## The function of non-histone crotonylation

### Protein activity

In addition to crotonylation of histones, the function of crotonylation of non-histones in various pathologic and physiological processes has attracted the attention of scientists. By using specific crotonylation antibody enrichment combined with high-resolution protein profiling, scientists found a large number of protonated modified proteins. Bioinformatics analysis found that nuclear proteins are enriched in crotonylation modification and participate in several physiological processes [43]. The biotinylated HDAC1 inhibits the deacetylase activity compared with the unmodified HDAC1, Sodium crotonate (NaCr) elevates the crotonylation of HDAC1. The p53 at ser46 was crotonylation after crotonic acid treatment, which led to the p53 activity inhibition in a dose-dependent manner. The crotonylation of p53 responds to the stress from the metabolism and DNA damage, which leads to the inhibition of p53 activity, elevates p53-dependent glycolytic activity, and enhances p53-dependent glycolytic activity [44].

### Protein localization

Recently, researchers have found that crotonylation influences the subcellular localization of proteins. HP1α is enriched in the heterochromatin by binding to methylated histones. The subcellular location of HP1α is mainly in the nucleus, where HP1 accumulates at heterochromatin regions and forms bright dots in Hela cells. The exposure of TSA or NaCr for 72 h, HP1α was reduced from heterochromatin and located in the nuclear matrix. Mechanically, the NaCr treatment elevates the crotonylation of HP1α, which results in the decreased binding activity to trimethylated H3K9 residues in vitro [43].

### Protein degradation

Ubiquitin-proteasome system (UPS) pathway and autophagy pathway are two main ways of protein degradation. The difference between the two is that cellular autophagy is thought to be involved in the degradation of most long-life proteins, as opposed to the ubiquitin-proteasome system, which mainly degrades short half-life proteins. UPS is the main pathway of protein degradation in cells and is involved in 80% of protein degradation in cells [45]. Recently, protein acylation was reported to be involved in the proteasome-dependent protein degradation. Crotonic acid influences the p53 degradation by elevating the crotonylation of p53. However, the pathway mediated with p53 degradation remains further studied [46]. The ubiquitin-proteasome-mediated proteolytic pathway is inhibited by acetylated modification. The mechanism for this phenomenon is that acetylation and ubiquitination share the same lysine sites, leading to competitive binding. Therefore, the acetylation maintains the stability of proteins. Given the structural similarity of acetylation and crotonylation, the study of the functions of crotonylation on protein degradation is pending further research.

## Crotonylation and related diseases

**Fig. 3:**
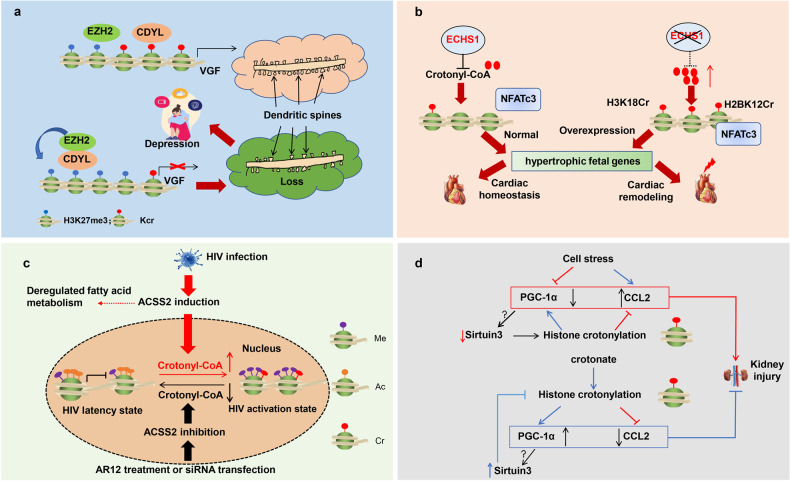
The functions of histone crotonylation in diseases. **a** Histone crotonylation regulates depression. CDYL influences synaptic plasticity by transcriptionally inhibiting the expression of neuropeptide VGF, thereby regulating the occurrence and development of stress-mediated depression. **b** Histone crotonylation elevates cardiac remodeling. ECHS1 hydrolyzes crotonyl-CoA, which maintains normal Kcr modification and expression of hypertrophic fetal genes. **c** The latency and activation of HIV are regulated by histone crotonylation. The reactivation of latent HIV depends on ACSS2 expression, and ACSS2 inhibition inhibits histone crotonylation–induced HIV replication and reactivation. **d** Histone crotonylation influences kidney injury. Histone crotonylation increases the expression of PGC-1α and SIRT3, decreases the expression of CCL2, and protects kidney function.

Crotonylation is one of the protein post-translational modifications, which regulates gene expression and subsequently influences the development and progress of diseases. We summarized the diseases related to crotonylation modification in Table 2. The regulatory mechanisms of abnormal crotonylation modification-related diseases are shown in Fig. 3.

**Table 2:** The diseases associated with crotonylation.

Diseases	Protein crotonylation	Mechanisms	Functions	Reference
Myocardial hypertrophy	H3K18Cr, H2BK12Cr	ECHS1 inhibition promotes H3K18Cr and H2BK12Cr modifications, elevates the recruitment of NFATC3 transcription factors on the promoter of myocardial hypertrophy gene.	The crotonylation results in cardiac remodeling	[49]
Myocardial I/R injury	IDH3a K199Cr, TPM1 K28/29Cr	Regulates apoptosis, cytoskeleton structure rearrangement, and cardiac function	The crotonylation promotes cardiac functions	[56]
Renal I/R injury	H3K9Cr	Crotonate increases the expression of renal PGC-1αand SIRT3, and decreases CCL2 though histone crotonylation	The crotonylation protects mice from renal I/R injury.	[30]
IgAN	Unknown	Regulates complement and coagulation cascades, and antigen processing and presentation pathways.	Unknown	[53]
Hemodialysis	Histone crotonylation	Crotonylated proteins are mainly enriched in the pathway associated with hemodialysis complications including complement and coagulation cascades, cardiac muscle contraction, and hematopoietic cell lineage.	Unknown	[54]
HIV lantency	Histone crotonylation	The histone crotonylation at LTR maintains HIV latency.	ACSS2-mediated H3K4 crotonylation promotes reactivation by elevating the levels of the crotonyl-CoA	[51]
Tumor development	Numerous histone and non-histone proteins crotonylation	P300 mediates the expression of HNRNPC by lysine crotonylation to enhance the proliferation, invasion, and migration of Hela cells.	Crotonylation accelerates the development of cancer	[18, 25, 55]
Depression	Histone crotonylation	CDYL inhibits the transcription activity of VGF and decreases structural synaptic plasticity.	The decreased Kcr modification leads to loss of dendritic spines	[47, 48]

## Histone crotonylation and depression

Depression is one of the most prevalent mental illnesses in modern society, affecting >300 million people worldwide. The occurrence of depression is not only regulated by genetics, but also closely related to environmental stress such as stress, traumatic memory, and other factors, but the molecular mechanism of environmental stress leading to depression is still unclear. It was found that the expression of a histone crotonylation hydrolase and a transcription suppressor CDYL significantly increased in the prelimbic cortex (PL) of depressed mice, and the level of crotonylation mediated by CDYL significantly decreased. When overexpresses CDYL in PL, it can increase the susceptibility of mice to depression. Conversely, when CDYL is knocked down, the susceptibility of mice to depression is reduced. High-throughput sequencing of RNA transcriptome showed that CDYL can affect synaptic plasticity by transcriptionally inhibiting the expression of neuropeptide VGF, thereby regulating the occurrence and development of stress-mediated depression [47, 48]. Mechanically, CDYL suppresses VGF expression through its dual effect on promoter histone Kcr and site-specific H3K27me3. In conclusion, the CDYL-VGF axis disrupts the structural synaptic plasticity in the mPFC, leading to behavioral depression (Fig. 3a).

## Histone crotonylation and cardiovascular disease

The heart is an energy-consuming organ, 70% to 90% of which comes from fatty acid metabolism. Fatty acid metabolism not only provides energy for cardiomyocytes but also produces many metabolites (such as crotonyl-coA, ketoglutaric acid, etc.) to modify histones, thus affecting the epigenetic inheritance and gene expression of cells. Apparent modification of histones plays an important role in maintaining cardiac homeostasis, among which crotonylation of histones (Kcr) is a new histone modification discovered recently. Enoyl-CoA hydratase short-chain 1 (ECHS1) is the downstream of SIRT3. The functions of ECHS1 are regulating the β-oxidation process of cellular fatty acids and hydrolyzing crotonyl-coA, which can be involved in the regulation of histone modification through the action of histone crotonyltransferase (HCT). Carriers of ECHS1 mutations in patients are prone to develop dilated cardiomyopathy and hypertrophic cardiomyopathy. Significantly reduced ECHS1 protein levels were found in the hearts of patients with heart failure. By constructing ECHS1 knockout mice, it was found that ECHS1 homozygous knockout mice produced embryo death, and heterozygous defective mice significantly promoted the occurrence of myocardial hypertrophy under the stimulation of angiotensin II. On the contrary, cardiomyocyte-specific overexpression of ECHS1 could protect against the occurrence of myocardial hypertrophy. Through RNA-seq high-through sequencing, biogenic analysis, and experimental studies, they found that ECHS1 defects enhanced histone H3K18cr and H2BK12cr modifications, promoted the recruitment of NFATC3 transcription factors on the promoter of myocardial hypertrophy gene, and promoted the occurrence of myocardial hypertrophy and cardiac remodeling [49]. This study suggests that the regulation of histone crotonylation is a new strategy for the treatment of cardiac hypertrophy and heart failure and has important clinical significance (Fig. 3b).

## Histone crotonylation and human immunodeficiency virus (HIV) latency

The stable viral reservoirs inhibit HIV eradication, and the HIV latency is epigenetic regulation [50]. Recently, histone crotonylation, a novel identified epigenetic modification of the HIV long terminal repeat (LTR), plays an important role in HIV latency. The presence of histone crotonylation is an important condition for latent HIV reactivation through elevating the levels of the crotonyl-CoA-producing catalyzed by ACSS2. The reprogramming of the HIV LTR at chromatin is enhanced by histone acetylation and inhibited by histone methylation HNRNPC. The HIV replication and reactivation are attenuated by histone crotonylation after pharmacologic inhibitor AR12 treatment or knockdown of ACSS2. The research found that the combination of ACSS2 overexpression with protein kinase C agonist (PEP005) or histone deacetylase inhibitor (vorinostat) reactivates latent HIV. In addition, the expression level of ACSS2 is increased and correlated with altered fatty acid metabolism in the intestinal mucosa of AIDS nonhuman primate model [51]. These results indicate that the maintenance of viral latency is related to histone crotonylation at the HIV LTR mediated by ACSS2 (Fig. 3c).

## Histone crotonylation and renal disease

Acute or chronic renal disease is involved in complex epigenetic mechanisms, in which crotylation is one of the important manner [25]. The cellular level of crotonic acid or crotyl-CoA regulates histone crotylation, and crotonate was applied to treat acute renal disease (Fig. 3d). The elevation of crotonylation and crotonate inhibits the inflammatory response as well as improves renal function. However, the accurate mechanism of crotylation in the regulation of renal injury remains for further study [52]. Acute renal injury is lethal to individuals without treatment other than kidney replacement. The level of histone H3k9cr is upregulated in the folic acid-induced acute renal tissue, which indicates that the inflammatory factors are involved in the modulation of histone crotonylation in kidney tubular cells [30]. The experiments of chromatin immunoprecipitation sequence indicate the enrichment of histone crotonylation at the gene encoding mitochondrial biogenesis regulator PGC-1α and the decrotonylase SIRT3 in acute renal injury tissue [30]. Crotonate elevates the expression of PGC-1α and SIRT3 and inhibits the level of CCL2 in kidney and renal tubular cell. What’s more, crotonate protects the mice against acute renal injury by elevating the level of PGC-1α and SIRT3 as well as inhibiting CCL2 [30]. Therefore, crotonate plays an important roles in protection of kidney damage by elevation of histone Kcr.

The immunoglobulin A nephropathy (IgAN) seriously impairs the kidneys’ ability to filter waste products from the blood, with the characterization of IgA deposition and deposition in the mesangial region with or without other immunoglobulins. The researchers discovered a lot of different expressed crotonylation proteins and modification sites in healthy persons and patients with immunoglobulin A (IgA) nephropathy by using the tandem MS and high-resolution liquid chromatography of crotonylation. The enrichment of crotonylated proteins in complement and coagulation cascades, and antigen processing and presentation pathways is an important element of IgA nephropathy revealed by the Kyoto Encyclopedia of Genes and Genomes (KEGG) and functional enrichment analyses [53].

Hemodialysis is a treatment for kidney failure by removing wastes and extra fluid from the blood. The researchers explore the crotonylation proteome by LC-MS/MS coupled with highly sensitive immune-affinity purification in normal people and hemodialysis patients, in which 1109 lysine modification on the 347 proteins was identified, and crotonylation of histone proteins was decreased in the renal failure patients undergoing hemodialysis. The result of KEGG-based functional enrichment analysis suggested that the biotinylated proteins are mainly enriched in the pathway associated with hemodialysis complications, including complement and coagulation cascades, cardiac muscle contraction, and hematopoietic cell lineage [54].

## Histone crotonylation and tumor development

The occurrence and development of tumors is a complicated process, in which protein modification is one of the key regulators. Recently, by using quantitative proteomics, the research has found that p300-mediated lysine crotonylation and p300-targeted Kcr substrate are involved in the regulation of cancer [18]. Crotonylation accelerates the development of cancer and is a carcinogenic factor. According to the exploration of the early detection research network EDRN database, 4.5% (20 out of 443) of cancer protein biomarkers were crotonylation. What’s more, 32 Kcr proteins are relevant to cancer genes and account for 5.9% of the total genes in the Catalog of Somatic Mutations in Cancer (COSMIC) gene database. The broad landscape of the Kcr pathway associated with tumor genes plays important roles in tumorigenesis and development [18]. HNRNPC, a high-degree protein node, has multiple p300-targeted sites and represents the sub-hubs of the p300-regulated protein interaction network. p300 enhances the proliferation, invasion, and migration of Hela cells by maintaining the level of heterogeneous ribonucleoprotein particle (HNRNPC) of lysine crotonylation [55]. What’s more, the expression of crotonylation is decreased in renal, liver, and gastric tissues but increased in colon, lung, thyroid, and pancreas carcinoma. Interestingly, the expression level of Kcr changes with the progression of liver cancer [55]. Apart from histone Kcr, the Kcr modification of non-histone also plays an important role in the progress of tumorigenesis. In the human lung cancer H1299 cell line, 2696 crotonylation sites were discovered in a total of 1024 proteins [25]. The biotinylated proteins are related to structural constituents of ribosomes, translation factor activity, and adenyl nucleotide binding revealed by gene ontology (GO) enrichment analysis [25]. These results reveal that non-histone crotonylation also plays an important role in tumorigenesis and development.

## Non-histone crotonylation and cardiovascular disease

**Fig. 4:**
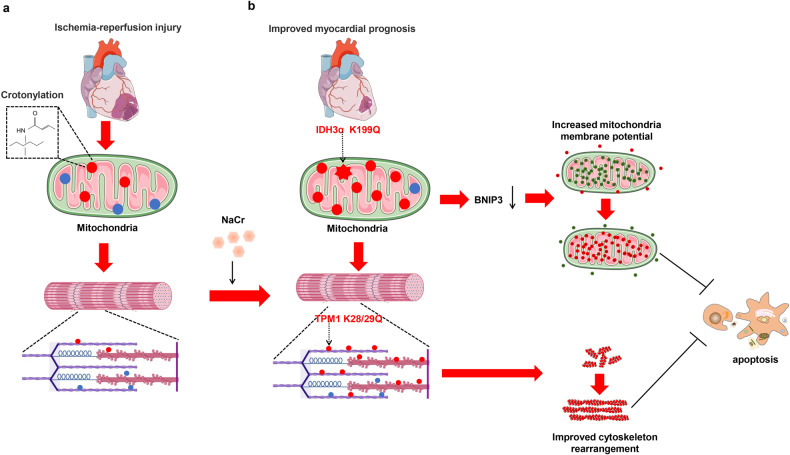
Non-histone crotonylation improved myocardial prognosis. **a** In the ischemia and reperfusion heart, low Kcr modification was discovered. **b** Crotonylation of IDH3α at K199 and the increasing crotonylation of TPM1 at K28/29 or enhancing general Kcr via NaCr supplement inhibits cardiomyocyte apoptosis by inhibiting BNIP3, which improves cardiac outcomes.

Crotonylation of lysine (Kcr) has recently been identified as a post-translational histone modification and is primarily involved in regulating a variety of nucleus-related cellular processes (Fig. 4). Recently, it has been reported that Kcr modification also exists in a large number of non-histone proteins. However, the role of Kcr modification, especially non-histone Kcr modification, in myocardial injury remains unclear. Protein modification group sequencing revealed that after cardiac ischemia-reperfusion injury (I/R), Kcr modification was significantly triggered and enriched in mitochondrial and skeleton proteins, which was completely different from that in other cells where Kcr modification mainly occurred in the nucleus (Fig. 4a). The changes of Kcr modification are related with the disruption of cardiomyocytes mitochondria, gap junction as well as cardiomyocytes autophagy and apoptosis. In addition, to mimic crotonylation, the researchers mutate the mitochondrial protein isocitrate dehydrogenase 3 [NAD+] alpha (IDH3α) at K199 and tropomyosin alpha-1 chain (TPM1) at K28/29 or treat cardiomyocytes with sodium crotonate to induce Kcr modification, which protects cardiomyocytes from apoptosis, promotes cytoskeleton structure rearrangement and boost heart function. Moreover, its protective mechanism is related to the regulation of mitophagy mediated by BNIP3 [56] (Fig. 4b).

## Conclusions and perspective

Protein acylation occurs in a variety of ways, including histone propionylation, malonylation, borylation crotonylation, and so on [50, 57–59]. Although protein acylation has been reported for many years, the concrete mechanism remains to be further studied [4]. Protein crotonylation has been recently reported as a histone marker of active promoter and is abundant in the male sperm cell [31]. However, the relationship between crotonylation and various human diseases is currently insufficient, and for which further research is needed. One of the focuses is the identification of the substrates of Kcr modification and their biological processes and functions regulated by this modification.

Crotonoyl-CoA as material for Kcr modification of histone and non-histone proteins regulates subsequent biological processes, which are mediated by ACSS2 and CDYL in the tissues and cells [60]. Thus, the content of crotonyl-CoA in tissue and subcellular compartments can reflect the occurrence of Kcr modification, and exploring the factors that influence crotonyl-CoA levels is also a promising area.

The enzymes that catalyze the modification and hydrolysis of Kcr still need further exploration [61, 62]. Besides, Kcr modification is similar to other modifications, such as acetylation modification in the catalytic system [63]. However, whether a specific enzyme for Kcr modification exists remains for further study. In addition, the identification of Kcr-specific enzymes will be a promising research field.

Kcr and other acylation modifications share some common modification enzymes, which raises the question of whether Kcr modifications have unique regulatory functions or redundant functions compared to other modifications. What’s more, the identification of different acylation modifications in the same lysine residue of proteins will be a significant research field.
